# Choriocarcinoma presenting as an isolated bone marrow metastasis—a case report

**DOI:** 10.3332/ecancer.2014.393

**Published:** 2014-01-29

**Authors:** Sarika Singh, Manjula Sardhana, Suneeta Sharma, Prakash Chitralkar

**Affiliations:** 1 Department of Pathology, Lady Hardinge Medical College, New Delhi, DL 110001, India; 2 Resident Pathology, Rajiv Gandhi Cancer Institute and Research Centre, New Delhi, DL 110085, India; 3 Medical Oncology, Rajiv Gandhi Cancer Institute and Research Centre, New Delhi, DL 110085, India

**Keywords:** bone marrow metastasis in choriocarcinoma, choriocarcinoma presenting as bone marrow metastasis, choriocarcinoma with bone marrow metastasis

## Abstract

Gestational trophoblastic diseases (GTD) are a heterogenous group of disorders that arise from trophoblastic epithelium. They can follow molar pregnancy (50%), abortions (25%), normal pregnancy (22.5%), and ectopic pregnancy (2.5%) [Robert JK, Lora HE, Brigitte MR (ed) Blaustein Pathology of the Female Genital Tract 6th edn (Springer) pp. 1101–7]. Occurrence of choriocarcinoma following normal delivery presenting as an isolated bone marrow metastasis is extremely rare, and no case has been cited in the literature, to the best of our knowledge. We are reporting a case of choriocarcinoma that presented with isolated bone marrow metastasis only.

## Introduction

Choriocarcinoma is rare, and it is one of the most malignant and aggressive neoplasms of all the gestational trophoblastic diseases (GTD). It can appear either after an intrauterine (normal) or an ectopic pregnancy, however, it is more common after a hydatidiform mole. Choriocarcinoma is associated with a high human chorionic gonadotrophin (HCG) level and rapid haematogenous spread to many organs. The most common sites for metastasis being the lung and vulvovaginal region followed by brain and liver. Other sites of metastasis, such as skin, gastrointestinal tract, kidney, breast, and bone are extremely rare. Presentation of choriocarcinoma as an isolated bone marrow metastasis is extremely rare, and no such case has been cited in the literature, to the best of our knowledge. Here, we report a case of choriocarcinoma that presented with bilateral toe gangrene and isolated bone marrow metastasis.

## Case report

**Figure 1.**
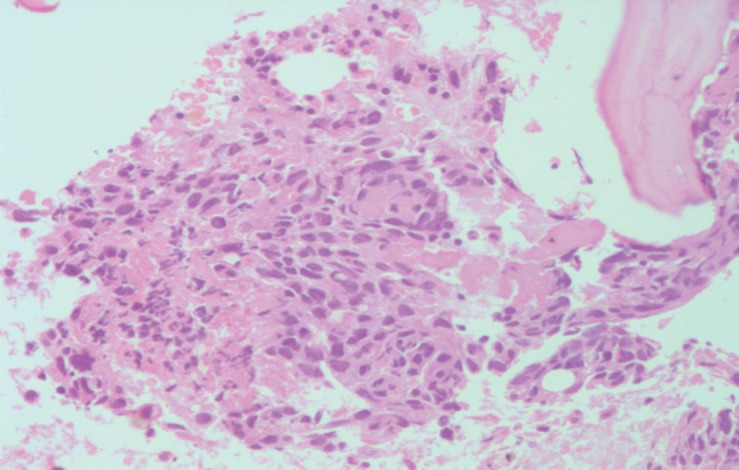
Bone marrow biopsy intertrabecular space showing bizarre multinucleated cells with hyperchromatic nuclei embedded in bed of necrosis and haemorrhage (200×) H&E.

**Figure 2.**
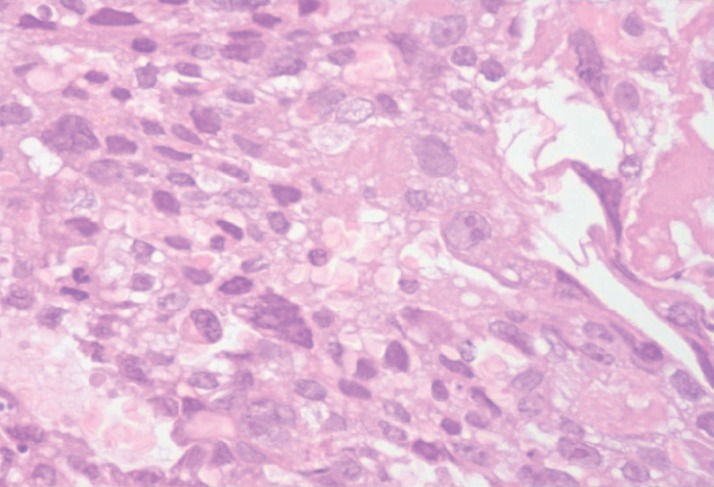
Tumour tissue showing predominantly polygonal cells with moderate to abundant clear cytoplasm, nucleus with high N/C ratio and coarse chromatin and prominent nucleoli (400×) H&E.

**Figure 3.**
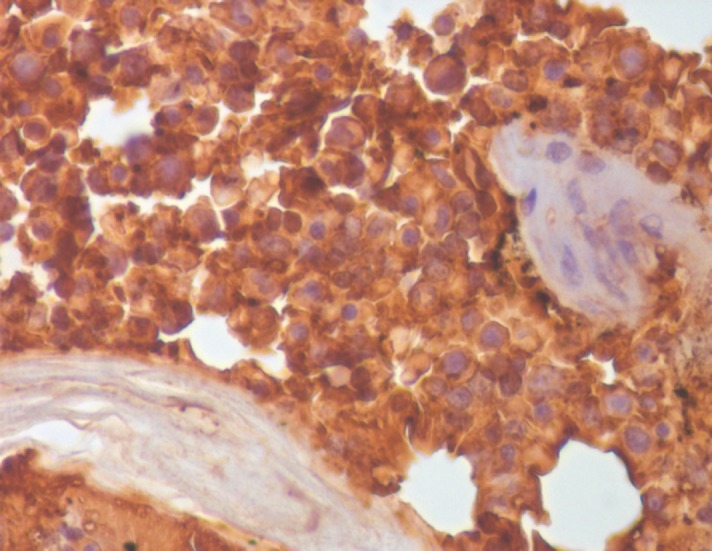
Tumour cells showing positive staining for βHCG on IHC (400×).

A 24-year-old primipara, who had a normal vaginal delivery four months previously, presented to us with complaints of fever off and on and oliguria. In the past, she had multiple episodes of vomiting, fainting, and giddiness for three months. An examination showed pallor, hepatomegaly, ascites, and bilateral second toe gangrene. The chest X-ray was within normal limits, the ultrasonography and computerised tomography scan showed a diffuse hypoechoic mass in the liver reported as a vascular lesion with a possibility of haemangioma or angiosarcoma. Based on these findings, the patient was suspected to have angiosarcoma. The routine blood examination revealed normocytic anaemia with neutrophilic leukocytosis. Subsequent to admission the patient developed coagulation derangement, hence limiting any liver biopsy (Table 1). However, a bone marrow examination was conducted prior to this and revealed erythroid hyperplasia, while a bone marrow biopsy showed extensive necrosis, bizarre cells, multinucleated cells, abnormal mitosis, and haemorrhage with normal haematopoietic areas interspersed in between (Figures 1 and 2). The possibility of choriocarcinoma/angiosarcoma was suggested. On Immunohistochemistry (IHC) tumour cells were positive for βHCG (Figure 3) while negative for CD31 and CK. Hence, a final diagnosis of choriocarcinoma metastatic to bone marrow was made. Ascitic fluid examination showed no malignant cells, however, there were extremely high βHCG levels (>3,000,000 mIU/μL). Following this report, the patient was subjected to meticulous gynaecological examination, since she had just delivered four months ago, and had experienced episodes of vomiting and fainting. She was made to undergo internal assessment (P/V examination), ultrasound, and CT scan whole examination, but no significant findings were observed. Unfortunately, by the time the case was worked up (three days), the patient succumbed to the disease before initiation of therapy and assessment of her serum βHCG levels.

## Discussion

GTDs encompass a varied group of neoplastic disorders that arise from the trophoblastic epithelium of the placenta, characterised by a distinct tumour marker (β-HCG) [1, 2]. GTDs are conventionally subclassified into five distinct groups on the basis of their histopathologic, cytogenetic, and clinical features. These groups are complete and partial hydatidiform mole, invasive mole, choriocarcinoma, and placental site trophoblastic tumour. Choriocarcinoma is a rare and highly malignant neoplasm of trophoblastic origin among the GTDs. This tumour is known for its association with molar pregnancy, a rapid haematogenous spread to multiple organs, high HCG levels and a good response to chemotherapy [2, 3]. It is preceded by several clinical conditions, it is observed that about 50% arise in molar pregnancies, 25% arise after previous abortions, 22.5% arise in normal pregnancies, and 2.5% arise subsequent to ectopic pregnancies [1–4].

Choriocarcinoma has an increased tendency to metastasise early by blood-borne dissemination, as in the present case. The favoured sites are the lung (94%), vagina (44%), liver (28%), and brain (28%), followed by the skin, gastrointestinal tract, kidney, breast, and bones [1, 5–7]. However, the isolated involvement of bone marrow is not cited in the literature. Approximately 30% of patients with choriocarcinoma show metastasis at the time of diagnosis [1, 8], generally associated probably with the higher affinity of trophoblasts to blood vessels. Because choriocarcinomas are very vascular lesions and are often perfused by fragile vessels, as well as due to the tendency of trophoblastic cells to invade and erode vessel walls, they are frequently haemorrhagic. Thus, intraparenchymal bleeding in any woman of child-bearing age with this malignancy is well explained [2, 9]. These lesions could be mistaken radiologically for hepatic vascular lesion as in the present case. The metastatic site being the presenting symptom in many cases, with the primary site being either too small or burnt out, often ruling out the diagnosis of choriocarcinoma [2, 5, 6, 10]. Thrombo embolic involvement is a known occurrence in choriocarcinoma both during therapy or after therapy [11–13]. The literature cites cases of gangrenous involvement of overlying scrotal skin in a case of mixed germ cell tumour of testis, but no case is cited in the literature with toe gangrene [14, 15]. Though in the present case the cause of this was not well elucidated, by conducting any Doppler or haemodynamic studies. There are cases of choriocarcinoma presenting as intra cerebral bleed with aneurysmal dilatation [12]. The proposed pathogenesis is not clearly understood but is probably due to the involvement of vessels because of the higher affinity of these malignant cells for blood vessels. Lepidini et al reported the development of both arterial and venous thrombosis in a case of testicular choriocarcinoma during chemotherapy. Proposed pathogenesis suggested by the author was an increased paraneoplastic stimulus of HCG, secondary to the marker surge phenomenon leading to hypercoagulability and thus subsequent thromboembolism [13]. Bony metastasis secondary to choriocarcinoma are very rare, as is evidenced by very few studies in [2, 5, 10]. Bone marrow involvement rather than bone is almost not heard of and therefore no citations appear in the literature to the best of our knowledge.

Bony metastasis with extra cortical compression being the alarming signs are usually picked up and managed. But with only bone marrow involvement, and no signs and symptoms suggestive of active bleeding present either at intraparenchymal or metastatic site, this case was missed and led to poor workup before it was referred to our centre. Thus, in a young female, episodes of postpuerperal giddiness, vomiting, and development of toe gangrene should have been an alerting chain of events, and intervention at the right time could have saved the patient, especially if compounded with assessment of s-β HCG levels.

## Conclusion

The aim of this case report is to raise awareness of this kind of rare presentation in young primiparae, since a high index of suspicion would have obviated the unfortunate demise of the patient, as this is a highly chemoresponsive malignancy.

## Figures and Tables

**Table 1.** List of investigations during patient’s stay in hospital.

	23/4 (Day1)	24/4 (Day2)	25/4 (Day3)	26/4 (Day4)	27/4 (Day5)
Hb (g/dl)		*7.7*	6.2	7.7	7.9
TLC (/mm^3^)		37.43 × 10^3^	40.68 × 10^3^	32.21 × 10^3^	23.82 × 10^3^
Blood urea (mg/dL)	36	59	97	114	128
S. creatinine (mg/dL)	0.8	1.5	2.2	2.6	2.4
S. sodium (meq/L)	127	127	125	123	127
S. potassium (meq/L)	3.5	5.2	5.5	5.4	4.3
S. uric acid (mg/dL)	7.3	10.1	13.7	12.8	9.9
S. direct bilirubin (mg/dL)	**2**	**3.4**	**4.9**		**13.6**
S. total bilirubin (mg/dL)	**3.8**	**5.2**	**8**		**23.3**
SGPT (U/L)	71	113	550		443
SGOT (U/L)	**398**	**921**	**6980**		**235**
S. alkaline phosphatase (U/L)	268	294	752		792
S. globulin (g/dL)	3.6	3.2	3.3		3
S. gamma GT (U/L)	52	45	59		79
APTT/PTTK (sec)			**77**	**84.6**	**61.1**
PT (s)			**37.7**	**33**	**19.2**
INR (s)			**4**	**3.40**	**1.77**
Serum CEA (ng/mL)		33.4			
Serum CA19.9 (u/mL)		117			
Serum AFP (ng/mL)		1.45			
Ascitic fluid β HCG				**30,000,000 mIU/uL**	
